# Preclinical characterization of a novel investigational monoclonal antibody CM313 with potent CD38-positive cell killing activity

**DOI:** 10.3389/fimmu.2024.1410457

**Published:** 2024-05-03

**Authors:** Wei Liu, Juntao Yu, Kaiwen Sun, Qin Song, Yuling Li, Yanyun He, Yanrong Wang, Gang Xu, Changyu Wang, Bo Chen

**Affiliations:** ^1^ Research and Development Department, Keymed Biosciences (Chengdu) Limited, Chengdu, China; ^2^ Department of Ecology and Evolutionary Biology, Tulane University, New Orleans, MS, United States

**Keywords:** CD38, CM313, preclinical, novel, monoclonal antibody

## Abstract

**Introduction:**

CM313 is currently under clinical investigation for treatments of multiple myeloma, systemic lupus erythematosus, and immune thrombocytopenia. We aimed to report the preclinical profile of the novel therapeutic anti-CD38 monoclonal antibody (mAb) CM313, with an emphasis on the difference with other CD38-targeting mAb.

**Methods:**

The binding of CM313 to CD38 recombinant protein across species was assessed using ELISA. The binding of CM313 to CD38-positive (CD38^+^) cells was detected using flow cytometry assays. CM313-induced complement-dependent cytotoxicity (CDC), antibody-dependent cellular cytotoxicity (ADCC), antibody-dependent cellular phagocytosis (ADCP) and apoptosis on different CD38^+^ cells were assessed by LDH release assays or flow cytometry assays. The effect of CM313 on CD38 enzymatic activity was measured using fluorescence spectroscopy. CM313 immunotoxicity in human blood was assessed using flow cytometry assays, ELISA, and LDH release assays. Anti-tumor activity of CM313 was assessed in multiple mouse xenograft models. Safety profile of CM313 were evaluated in cynomolgus monkeys and human CD38 transgenic (B-hCD38) mice.

**Results:**

There exist unique sequences at complementarity-determining regions (CDR) of CM313, which facilitates its affinity to CD38 is consistently higher across a spectrum of CD38^+^ cell lines than daratumumab. In vitro studies showed that CM313 induces comparable killing activity than daratumumab, including ADCC, CDC, ADCP, apoptosis induced by Fc-mediated cross-linking, and effectively inhibited the enzymatic activity of CD38. However, CM313 showed more potent CDC than isatuximab. *In vivo*, CM313 dose-dependently inhibited xenograft tumor growth, both as a monotherapy and in combination with dexamethasone or lenalidomide. Furthermore, CM313 was well tolerated with no drug-related clinical signs or off-target risks, as evidenced by 4-week repeat-dose toxicology studies in cynomolgus monkeys and B-hCD38 mice, with the later study showing no observed adverse effect level (NOAEL) of 300mg/kg once weekly.

**Discussion:**

CM313 is a novel investigational humanized mAb with a distinct CDR sequence, showing comparable killing effects with daratumumab and stronger CDC activity than isatuximab, which supports its clinical development.

## 1 Introduction

CD38 (cluster of differentiation 38) is a type-II transmembrane glycoprotein that functions both as a receptor and an ectoenzyme (1, 2). As a receptor, CD38 interacts with the ligand CD31 to regulate lymphocyte migration, proliferation, and differentiation (3). In addition, CD38 ecto-enzymatic activity leads to syntheses of cyclic adenosine diphosphate ribose (cADPR) from nicotinamide adenine dinucleotide (NAD^+^), as well as nicotinic acid adenine dinucleotide phosphate (NAADP) from nicotinamide adenine dinucleotide phosphate (NADP^+^), thereby regulating calcium signaling (4, 5) and contributing to the formation of immunosuppressive microenvironment (6). CD38 is absent on early hematopoietic progenitors, while its expression is broadly distributed at steady-state on both myeloid and lymphoid cells (7) including B cells, T cells, natural killer (NK) cells, innate lymphoid cells (ILC), monocytes, dendritic cells (DC), and monocytes (8). The pattern of CD38 expression during cell cycles of these immune cells suggests that the impact of CD38 may encompass a range of processes, from development, activation to suppression. Specifically, the role of CD38 is most extensively discussed in B cell lineage, where elevated CD38 expression in plasma cells is directly related to the secretion of pathologic autoantibodies. It has also been reported that CD38 appears to regulate CD8+ T cells, NK cells and DCs influencing various aspects such as cytokine release, adhesion, and cellular migration toward sites of inflammation (9–12). Given powerful functions CD38 possessed during multiple cell fate-determining stages, it is recognized as an emerging therapeutic target under conditions in which cellular metabolism is altered to trigger infection, aging, and tumorigenesis.

Initial practices of anti-CD38 therapies targeted against hematologic malignancies, a highly heterogeneous group of diseases that originate in the blood, bone marrow, and lymphatic system (13). Multiple myeloma (MM), the second most common hematologic malignancy, is characterized by a clonal expansion of aberrant plasma cells in the bone marrow that invade the bone and other organs, resulting in end-organ damage (14). First-line treatment for MM typically involves a combination of a proteasome inhibitor, an immunomodulatory drug (IMiD) and/or dexamethasone (15). However, relapse and resistance can occur even after complete remission (16, 17), and there is still a demand for approaches that enable long-term tumor regression. Antibody-based immunotherapy, with its high target affinity and specificity, offering superior and durable treatment outcomes with manageable side effects, has been considered to be a mainstay treatment option for patients alongside chemotherapy, surgery and radiation (18). For effective targeting, qualified therapeutic antibodies are expected to possess both cytostatic and cytotoxic capabilities, along with high target expression on tumor cells. In this regard, CD38 that selectively expressed in pathologic MM cells, represents an ideal target (19, 20). To date, the FDA-approved anti-CD38 monoclonal antibodies (mAbs), daratumumab (Darzalex, Janssen) and isatuximab (Sarclisa, Sanofi), have demonstrated encouraging anti-MM activity with manageable safety profiles for both relapsed/refractory multiple myeloma (RRMM) and newly diagnosed MM (21, 22).

On the other hand, the current predominant focus of CD38-targeting treatment lies in the field of autoimmune diseases (AIDs). The pathologies of AIDs have been extensively investigated to favor mechanism of action (MOA) of anti-CD38 mAb, featured with potency to reduce autoantibody secretion via CD38-positive (CD38^+^) cells depletion. For example, applying daratumumab in the treatment of immune thrombocytopenia (ITP), neuromyelitis optica (NMO) and systemic lupus erythematosus (SLE) included AIDs has been testified by global clinical trials at stages of phase II or phase III (ITP, NCT04703621; NMO, NCT05403138; SLE, NCT04810754) (23, 24). Despite increasing numbers of active clinical trials following this trend, complicated pathologies related to induction, progressions, and reoccurrence of diverse types of AIDs set high demands for novel drugs targeting CD38.

In this context, we developed a new anti-CD38 mAb, namely CM313, with unique sequences in CDRs compared with other molecules. We characterized the MOA of CM313 in several preclinical models of CD38^+^ cell lines, with its safety testified in cynomolgus monkeys and human CD38 transgenic (B-hCD38) mice. Currently, CM313 is under clinical trials in phase I for the treatment of RRMM (NCT04818372), phase I/II for SLE (NCT05465707), and phase II for ITP (NCT05694767).

## 2 Materials and methods

### 2.1 Antibodies generation

The humanized antibody CM313 against human CD38 was generated using hybridoma technology. Briefly, female BALB/c mice aged 6-8 weeks were immunized with recombinant CD38 protein mixed 1:1 with TiterMaX adjuvant to form an emulsion. Immunizations were performed every two weeks for a total of four times. The initial immunization dose was 50 μg per mouse, and the dose was subsequently reduced to 25 μg. After four immunizations, mice with favorable serum titers were selected for a final immunization with 10 μg CD38 protein. Following immunization of BALB/c mice with recombinant human CD38 antigen, splenocytes were harvested and fused with SP2/0 myeloma cells. 576 clones were identified to binding specifically to the recombinant CD38 protein. Next, 83 positive clones binding with CD38-expressing cells were identified using flow cytometry. These clones were further screened for their capacity to mediate CDC activity. The leading candidates underwent computer-assisted humanization to enhance their therapeutic potential. The humanized antibodies were then subjected to rigorous evaluation of their CDC, ADCC, and ADCP activities against a panel of CD38-positive cell lines, including Ramos, TALL-1, Daudi, and NALM-6. This comprehensive assessment resulted in the selection of CM313 as the final candidate molecule for further development.

### 2.2 Binding of CM313 to recombinant CD38 protein across species

100 μL of 1 μg/mL recombinant CD38 proteins from human, chimpanzee, cynomolgus monkey, marmoset, rat, mouse, rabbit and dog were encapsulated in 96-well enzyme-labeled plates using enzyme-linked immunosorbent assay (ELISA). After blocking with 5% skimmed milk, serial dilutions of CM313 or isotype control were added. The horseradish peroxidase (HRP)-labelled goat anti-human secondary antibody was then used to assess the binding of CM313 to different species of CD38, with absorbance was measured at 450 nm. The antibody concentration-absorbance (A_450_) curve was obtained by fitting the four-parameter model of absorbance, and the half effective binding concentration (EC_50_) of CM313 was calculated.

### 2.3 Binding of CM313 to CD38^+^ cells

CD38^+^ cells including multiple myeloma cell lines (RPMI 8226, NCI-H929, MM.1S), Burkitt’s lymphoma cell lines (Ramos, Daudi, Raji), diffuse large B lymphoma cell line (Toledo), acute B lymphoid leukemia cell lines (RS4;11, NALM-6), and acute T lymphoid leukemia cell lines (CCRF-CEM, TALL-1, HuT 78) were obtained from American Type Culture Collection. Cells were cultured in RPMI 1640 supplemented with 10% fetal bovine serum and 2 mM L-Glutamine (HyClone) at 37°C in a humidified 5% CO_2_ incubator. 5×10^4^ tumor cells were incubated with serial dilutions of CM313 or isotype control for 45 min. After washing and incubation with Alexa Fluor 647-conjugated goat anti-human IgG (Jackson ImmunoResearch) for a further 45 min, the cells were resuspended in propidium iodide (PI). Cell-associated fluorescence was analyzed using a FACSCelesta with FACSDiva™ software (BD Biosciences).

### 2.4 Antibody-dependent cellular cytotoxicity assay

5×10^4^ target cells (NCI-H929, MM.1S, Daudi, Toledo, RS4;11, NALM-6, CCRF-CEM, HuT 78) and NK-92MI-CD16a cells were co-incubated at a target: effector (E: T) ratio of 1:2 with serial dilutions of CM313 or isotype control, respectively. After 4-hour incubation, the release of lactate dehydrogenase (LDH) in the supernatant was measured using the CytoTox-ONE Homogeneous Membrane Integrity Assay Kit (Promega, G7891) according to standard protocols. The degree of specific lysis was calculated as follows: % lysis= (experimental release - spontaneous release)/(maximum release - spontaneous release) * 100%.

### 2.5 Complement-dependent cytotoxicity assay

5×10^4^ Ramos or CHO-hCD38 cells (CHO cells with stable overexpression of human CD38) were used as target cells and incubated with serial dilutions of CM313 or isotype control at 5% normal human serum complement for 5 hours. The released LDH was measured using CytoTox 96^®^ Non-Radioactive Cytotoxicity Assay Kit (Promega, G1780) to calculate the viability of the target cells, with calculation method as above.

### 2.6 Antibody-dependent cellular phagocytosis assay

5×10^4^ Daudi or NCI-H929 as target cells were labeled with the live cell fluorescent dye carboxyfluorescein diacetate succinimidyl ester (CFSE, 10 nM), and the labeled target cells and macrophages were mixed and co-incubated at a ratio of 4:1 with serial dilutions of CM313 or the isotype control for 4 hours. The effector macrophages were isolated and differentiated from peripheral blood of healthy volunteers. After CD14^+^ mononuclear cells were isolated and purified from human peripheral blood mononuclear cells (PBMC), they were induced to differentiate into macrophages as effector cells by culture. Flow cytometry was used to calculate the rate of phagocytosis by detecting the CD14^+^CFSE^-^ macrophages (without endocytosis) and CD14^+^CFSE^+^ macrophages (with endocytosis).

### 2.7 Induction of apoptosis by Fc-mediated cross-linking

1.4×10^6^ Ramos cells were incubated with 100 μL of 66.7 nM CM313 or isotype control in the presence or absence of goat anti-human IgG Fcγ fragment for 24 hours. In addition, 5 μL of 1 mM camptothecin was used as a positive control to induce apoptosis for 4 hours. Cells were then stained with FITC-labeled annexin V and PI. The percentage of apoptotic cells was determined using flow cytometry, detecting annexin V^+^PI^-^ cells (early apoptotic cells) and annexin V^+^PI^+^ cells (late apoptotic cells).

### 2.8 CD38 enzymatic activity assay

1×10^5^ CHO-hCD38 cells were incubated with 100 nM CM313 or isotype control for 15 min. 50 μL of 80 μM nicotinamide guanine dinucleotide (NGD^+^), a structural analog of NAD^+^, was then added as a substrate of CD38 at 0 min, 5 min, 9 min, 15 min, 30 min, 60 min, 90 min, 120 min, and 156 min. The relative fluorescence unit (RFU) of the catalysate, cyclic guanine diphosphate ribose (cGDPR), was measured to determine the relative inhibition of enzyme activity. Inhibition rate (%) = (experimental RFU - isotype RFU)/isotype RFU * 100%.

### 2.9 Mouse tumor xenograft models

40 female CB-17 SCID mice aged 5-7 weeks and 160 BALB/c nude mice aged 4-5 weeks were used for tumor fragment implantation. Mice were obtained from Beijing Vitonglihua Laboratory Animal Technology Co., LTD and Beijing Huafukang Biotechnology Co., LTD, respectively. All mice were maintained under the specific pathogen free (SPF) level according to the guidelines of the Association for Assessment and Accreditation of Laboratory Animal Care International (AAALAC). The health and mortality of the mice were evaluated daily, with clinical signs of illness and discomfort were examined at least twice a week. The maximal tumor size permitted by ethics committee/IRB was set as 1500 mm^3^ for Daudi and RPMI 8226, and 2500 mm^3^ for MM.1R tumor xenograft models. All results were confirmed not to exceed the maximal tumor size.

#### 2.9.1 Daudi tumor xenograft model

1.8×10^7^ Daudi cells were injected subcutaneously into each CB-17 SCID mouse. When tumor volume reached 100-150 mm^3^ (referred to as day 0), mice were divided into groups based on tumor volume (N= 10/group). CM313 (0.3, 1, 3 mg/kg) was administered intravenously in a volume of 10 mL/kg body weight on day 0. Anti-KLH hIgG1 (3.0 mg/kg) was administered intravenously as a control.

#### 2.9.2 MM.1R tumor xenograft model

2×10^7^ MM.1R cells were injected subcutaneously into each BALB/c nude mouse. When tumor volume reached 100-150 mm^3^ (referred to as day 0), mice were divided into groups based on tumor volume (N= 10/group). CM313 (0.3, 1, 3 mg/kg) was administered intravenously on days 0 and 3. Moreover, a combination treatment involving 1 mg/kg dexamethasone (intraperitoneal, days 0-13) and 0.3 mg/kg CM313 was performed in MM.1R tumors. Anti-KLH hIgG1 (3.0 mg/kg) was administered intravenously as a control.

#### 2.9.3 RPMI 8226 tumor xenograft model

RPMI 8226 tumor xenograft model was used to detect the effect of the combination of CM313 and lenalidomide. 2×10^7^ RPMI 8226 cells were subcutaneously injected into each BALB/c nude mouse. When the tumor grew to 100-150 mm^3^ (referred to as day 0), mice were divided into groups based on tumor volume (N= 10/group). CM313 (3, 7, 15 mg/kg) was administered intravenously on days 0, 3, 6, 10, and 13. Moreover, a combination strategy involving 10 mg/kg lenalidomide (intragastric, days 0-20) and 3 mg/kg CM313 was implemented. Anti-KLH hIgG1 (15.0 mg/kg) was administered intravenously as a control.

At the end of the experiment, the mice were euthanized using a CO_2_-rich cage and the tumors were then dissected and photographed. All mice survived to the end of the experiment and were used for further investigations. The experimental index was to examine the effect of the drug on tumor growth in terms of T/C (%) or tumor growth inhibition (TGI) (%). The tumor diameter was measured twice a week with vernier calipers, and the tumor volume (V) was calculated by the following formula: V=1/2 * a * b^2^, where a and b denoted the length and width, respectively. T/C (%) = (T-T_0_)/(C-C_0_) * 100%, where T and C are the tumor volumes of the experimental and control groups at the end of the experiment; T_0_ and C_0_ are the tumor volumes at the beginning of the experiment. TGI (%), tumor growth inhibition (%) = 100- T/C (%).

### 2.10 Preclinical toxicity and safety in cynomolgus monkeys and B-hCD38 mice

Cynomolgus monkeys received repeat intravenous administration of CM313 (50 and 206 mg/kg) once a week for 4 weeks (5 doses). B-hCD38 mice received repeat intravenous administration (300 mg/kg) of CM313 once a week for 4 weeks (5 doses). The parameters were assessed as follows: morbidity and mortality, clinical observations, body weights, food consumption, body temperature, electrocardiograms examination, clinical pathology (hematology, coagulation, clinical chemistry) and gross pathology, as well as toxicity, drug exposure, immunotoxicity and immunogenicity of CM313. The serum concentrations of CM313 and anti-CM313 antibodies (ADAs) were measured by the validated ELISA analytical methods. The lower limit of quantitation of CM313 and ADAs were 100 ng/mL.

### 2.11 Detection of CM313 immunotoxicity in human blood

The studies involving human samples were approved by Shanghai Liquan Hospital ethics committee (20212002-C221).

#### 2.11.1 Binding to human blood cells

Peripheral venous blood was collected from healthy volunteers. To assess CM313 binding to subpopulations of human blood, 45 μL of blood was mixed with 5 μL of Alexa Fluor 488-labelled CM313 or isotype control, along with 1 μL of cell-specific antibodies for 30 minutes. Flow cytometry was employed to evaluate CM313 binding to distinct subpopulations of human blood. The following antibodies were utilized for characterization: CD3-Brilliant Violet 421 (Biolegend, 317344), CD19-P-phycoerythrin (Biolegend, 302254), and CD14-allophycocyanin (Biolegend, 301808). Erythrocytes and granulocytes were distinguished based on cell size (FSC-A) and cell granularity (SSC-A). BD FACSCelestaTM Cell Analyzer (BD BioSciences) was used to perform flow cytometry in the detection of CM313 immunotoxicity in human blood, and the data was analyzed using FlowJo V10 software.

#### 2.11.2 Binding to human platelets

To examine binding to human platelets, platelet suspensions were isolated via 3.8% sodium citrate. Following centrifugation, the platelet suspension was blocked with ChromPure mouse IgG for 10 minutes on ice. Subsequently, 50 μL of platelet suspension was mixed with 50 μL of 200 μg/mL CM313 or isotype control, and incubated for 45 minutes on ice at room temperature. Flow cytometry, using the anti-human IgG-AF647 antibody, was employed to detect CM313 binding to human platelets.

#### 2.11.3 Assessment of hemolysis by CDC

Freshly isolated human peripheral blood erythrocytes were used as target cells to evaluate whether binding to erythrocytes leads to hemolysis by CDC. Ramos cells and cell lysis were used as positive controls. 1×10^6^ erythrocytes or 1×10^8^ Ramos cells were co-incubated with CM313 (100 and 10 μg/mL) or the isotype control in the presence of 5% normal complement for 5 hours. The released LDH was measured using CytoTox 96^®^ Non-Radioactive Cytotoxicity Assay Kit (Promega, G1780).

#### 2.11.4 Risk of cytokine release syndrome assessment

Peripheral blood was collected from six healthy volunteers to assess the potential for CRS. CM313 (100 and 10 μg/mL) were co-incubated with the blood for 24 hours, and anti-CD3 (OKT3) antibody was used as a positive control, Anti-KLH hIgG1 was used as a negative control. Serum levels of six cytokines, including interleukin 2 (IL-2), interleukin 4 (IL-4), interleukin 6 (IL-6), interleukin 1β (IL-1β), tumor necrosis factor α (TNF-α) and interferon γ (IFN-γ), were quantified using the multi-specimen flow protein quantification technique (Cytometric Bead Array).

### 2.12 Statistical analysis

GraphPad Prism 9.0 software was used for statistical analysis. The tumor volume (mean and standard error) of indicated groups were analyzed through the two-tailed Student’s *t* test. A p-value of < 0.05 was considered statistically significant.

## 3 Results

### 3.1 Binding of CM313 to CD38

**Figure 1:**
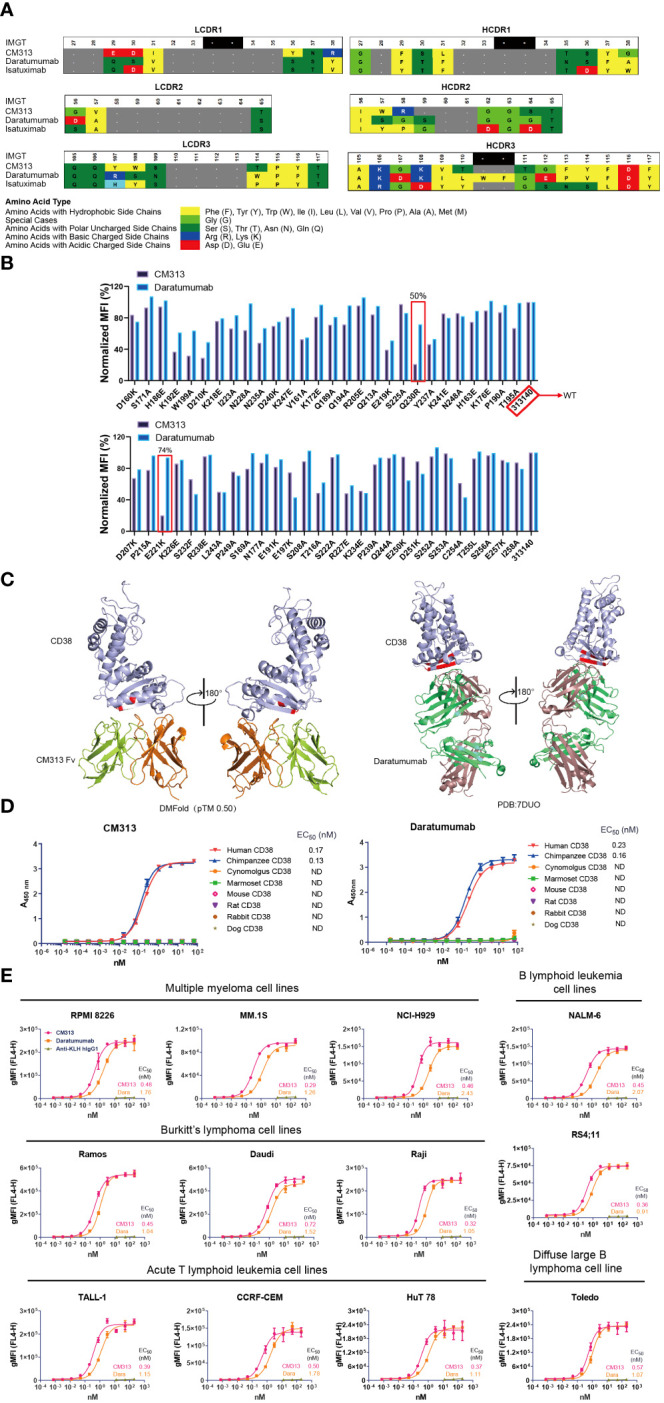
Binding affinity of CM313 to CD38. **(A)** Binding of CM313 to CD38 from different species was assessed using ELISA. EC^50^, Half-maximal effective concentration. ND, Not detected. **(B)** Binding of CM313 and daratumumab to different CD38 mutants. **(C)** 3D visualization of epitope differences on the human CD38 molecule recognized by CM313 (DMFolD) and daratumumab. **(D)** Binding of CM313 to CD38 from different species. **(E)** Binding of CM313 to CD38-positive cells. CD38-positive (CD38^+^) cell lines were incubated with CM313 or Anti-KLH hIgG1, followed by flow cytometry analysis. Binding intensity was presented as geometric mean fluorescence intensity (gMFI).

Among various sources of CD38-targeting mAbs, the newly generated CM313 (IgG1κ form) is a distinctive competitor featured with an innovative molecular structure. Specifically, at both heavy and light chain complementarity-determining regions (HCDR, LCDR) 1-3 of CM313, several amino acids at the same locations exhibit different properties compared to other CD38 mAbs, such as daratumumab (Darzalex, Janssen) and isatuximab (Sarclisa, Sanofi) (**Figure 1A**). Mutational analysis also indicates that CM313 and daratumumab bind to distinct epitopes on human CD38. Specifically, the CD38 mutants Q230R and E221K significantly impair the binding of CM313 but not daratumumab (**Figure 1B**). Additionally, 3D visualization reveals differences in the epitope on human CD38 recognized by CM313 and daratumumab (**Figure 1C**).

Given the structural differences, we are leaning towards functional uniqueness of CM313. The affinity of CM313 to CD38 across species was assessed first, using ELISA to detect its binding to the recombinant CD38 proteins from cynomolgus, marmoset, mouse, rat, rabbit and dog. The results showed that, similar to the FDA-approved daratumumab, CM313 only bound human and chimpanzee CD38 with high affinity and the half maximal effective concentration (EC_50_) was 0.17 nM and 0.13 nM, respectively (**Figure 1D**).

Furthermore, to validate the affinity of CM313 to the native conformational structure of CD38, CD38^+^ cells were used as binding targets of CM313 in flow cytometry. As shown in **Figure 1E**, the EC_50_ values of CM313 to target cell lines ranging from 0.29 to 0.72 nM, lower than those of daratumumab, which varied from 0.91 to 2.43 nM. Hence, CM313 exhibited robust and dose-dependent target engagement, with potent binding affinity across CD38^+^ cell lines even higher than daratumumab.

### 3.2 Effect of CM313 on cytotoxicity and CD38 enzyme activity *in vitro*

**Figure 2:**
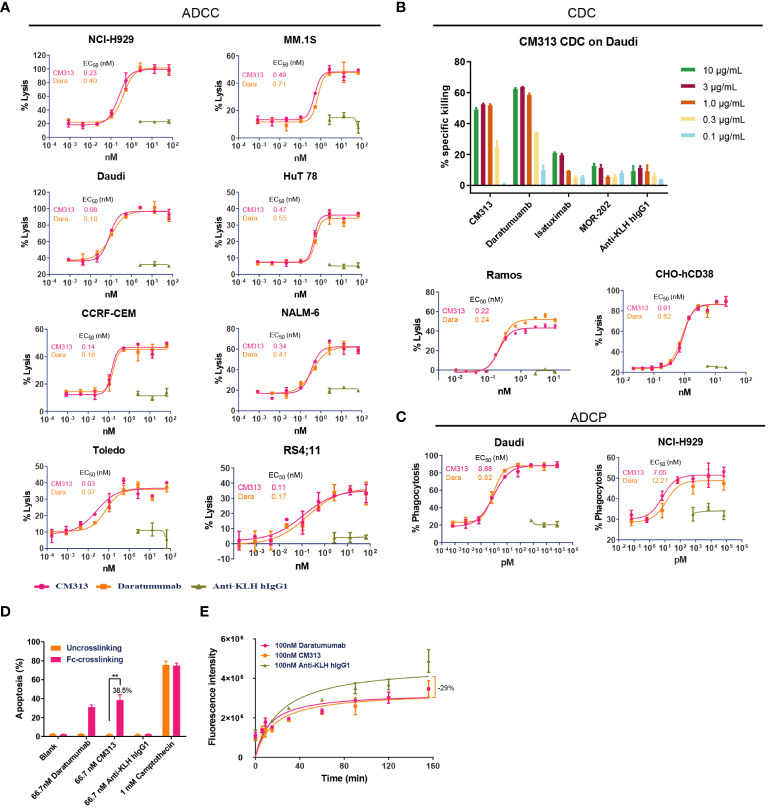
Multiple tumor cell killing activity of CM313 *in vitro*. **(A)** Dose-dependent ADCC induced by CM313. **(B)** Dose-dependent CDC induced by CM313. **(C)** Dose-dependent ADCP induced by CM313. The percentage of phagocytosis was detected by measurement of the percentage of CD14^+^CFSE^+^ macrophages by flow cytometry and presented as mean and standard deviation. **(D)** Apoptosis induced by Fc-mediated cross-linking. The percentage of apoptosis was detected by measurement of the percentage of annexin V^+^PI^-^ cells and annexin V^+^PI^+^ cells by flow cytometry and presented as mean and standard deviation. **p <0.01, between the indicated groups. **(E)** Inhibition of CD38 enzymatic activity.

As an IgG1 antibody, CM313 possesses the potential to induce ADCC, CDC and ADCP via its IgG constant domains (Fc domain) (7, 25). Consequently, *in vitro* experiments were performed to assess CM313’s immunoglobulin-mediated cytotoxicity. Upon binding to CD38, CM313 exhibited dose-dependent ADCC activity with EC_50_ values ranging from 0.03 nM to 0.49 nM and maximal lysis ranging from 35-95%, as measured by the release of LDH in the supernatant in the indicated cell lines (**Figure 2A**). Remarkably high ADCC activities (maximal lysis >90%) were observed in NCI-H929 and Daudi cells (**Figure 2A**). Overall, CM313 showed similar ADCC killing potency with daratumumab. Next, we test CDC activity of CM313 with other anit-CD38 agents, and the results showed CM313 comparable CDC activity with daratumumab, but more potent than isatuximab and MOR-202 (**Figure 2B**). Additionally, CM313 showed dose-dependent ADCP activity (**Figure 2C**). To gain comprehensive insights into CM313’s mechanism of action, we investigated the phenomenon of apoptosis induced by Fc-mediated cross-linking, another pathway for eliciting tumor cell death (26). A significant amount of apoptosis was observed when the Fc cross-linking condition was applied, with an apoptosis rate of 38.5% (**Figure 2D**).

In addition to immunoglobulin-mediated cytotoxicity, CM313 binding to CD38 may also affect the ecto-enzymatic activity of CD38. The ecto-enzymatic activity of CD38 leads to the synthesis of cADPR from NAD^+^ and also the production of extracellular adenosine, which may contribute to immune evasion of tumor cells (6). To determine the inhibition of CD38 ecto-enzymatic activity by CM313, CHO-hCD38 cells were co-incubated with 100 nM CM313 and NGD^+^, an NAD^+^ analog. CM313 showed partial inhibition of CD38 enzymatic activity, with maximum inhibition rate of 29% when compared to the isotype control (**Figure 2E**).

In summary, CM313 elicits immune responses, including ADCC, CDC, ADCP, and apoptosis induced by Fc-mediated cross-linking, to target and eliminate tumor cells. Additionally, CM313 exhibits inhibitory effects on CD38’s extracellular enzymatic activity. To be noticed, among all the evaluations accomplished above, CM313 exerted comparable killing activity with daratumumab, but more potent CDC than isatuximab.

### 3.3 CM313-mediated anti-tumor activity in mouse xenograft models

**Figure 3:**
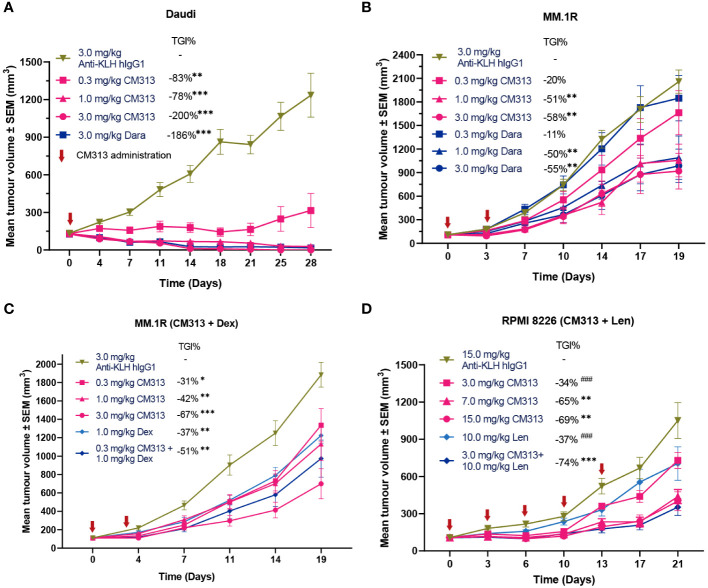
CM313 induces tumor regression as monotherapy and in combination with dexamethasone or lenalidomide in mouse models. **(A)** Daudi cell xenograft. CM313 was intravenously injected as indicated. ^**^ *p <*0.01, ^***^ *p <*0.001, compared with Anti-KLH hIgG1 group on day 28. **(B, C)** MM.1R cell xenograft. CM313 was injected intravenously alone **(B)** or in combination with dexamethasone **(C)**. Dexamethasone was injected intraperitoneally from day 0 to day13. ^*^ *p <*0.05, ^**^ *p <*0.01, ^***^ *p <*0.001, compared with Anti-KLH hIgG1 group on day 19. **(D)** RPMI 8226 cell xenograft. CM313 was injected intravenously in combination with lenalidomide. Lenalidomide was injected intragastrically from day 0 to day 20. ^*^ *p <*0.05, ^**^ *p <*0.01, ^***^ *p <*0.001, compared with Anti-KLH hIgG1 group on day 21; ^###^ *p <*0.001, compared with 3.0 mg/kg CM313 + 10.0 mg/kg group on day 21. Dara, daratumumab; Dex, dexamethasone; Len, lenalidomide; TGI%, tumor growth inhibition (%). Tumor volume was presented as mean and standard error. Anti-KLH hIgG1 was used as an isotype control.

To investigate whether these aforementioned cytotoxic effects of CM313 translate into anti-tumor efficacy *in vivo*, we evaluated its efficacy in multiple cell line-derived xenograft models via subcutaneous inoculation of tumor cell lines including Daudi lymphoma, MM.1R and RPMI 8226 multiple myeloma. Single-dose administration of CM313 (0.3, 1, 3 mg/kg) resulted in dose-dependent inhibition of subcutaneously transplanted Daudi tumors, achieving reduction of 83%, 178% and 200%, respectively (**Figure 3A**), in which case we also analyzed and found CM313 achieved similar tumor reduction with daratumumab. Similar outcomes were observed in MM.1R tumors (**Figure 3B**).

Notably, CD38-targeting mAbs, often used in combination with dexamethasone and/or lenalidomide, have been approved for the treatment of MM (17). Hence, we further evaluated the anti-tumor activity of CM313 in combination with dexamethasone or lenalidomide administration. CM313 (0.3 mg/kg) plus dexamethasone (1 mg/kg) increased the tumor growth inhibition rate to 51%, compared to 37% for dexamethasone alone (**Figure 3C**). In addition, CM313 (3 mg/kg) plus lenalidomide (10 mg/kg) increased the tumor growth inhibition rate to 74%, significantly surpassing the effects of either drug alone (**Figure 3D**). The tumor-bearing mice exhibited favorable tolerance to all drug treatments, with no discernible weight loss symptom.

### 3.4 Preclinical toxicity of CM313

As mentioned above, similar to the anti-CD38 mAb drugs daratumumab (Darzalex, Janssen) and isatuximab (Sarclisa, Sanofi), CM313 only bound CD38 sourced from human and chimpanzee, both inapplicable for safety evaluation. We followed the same strategy that isatuximab applied for FDA approval and used cynomolgus monkeys to validate its safety of the CM313 formulation. Results of the 4-week repeat-dose toxicology study suggested that CM313 was well tolerated when administered once weekly to cynomolgus monkeys in both 50 and 206 mg/kg groups. No unexpected death, drug-related clinical sign, or weight loss was observed during the study. Drug exposure (C_max_ and AUC_0–168h_) increased in approximate proportions to the doses on day 1 and day 22. Besides, no sex-specific difference in exposure between males and females. The immunogenicity assay of CM313 showed no ADA detection in cynomolgus monkeys after repeated dosing. Under such circumstances where no conventionally defined relevant species existed, we used CD38 humanized mice with a C57BL/6N background, namely B-hCD38 mice, to initially evaluate the toxicological properties of CM313. B-hCD38 mice of both sexes were intravenously injected once a week for 4 weeks (5 doses). The no observed adverse effect level (NOAEL) dose was 300 mg/kg with mean AUC_last_ of 445000 and 554000 h·μg/mL for the last dose in female and male mice, respectively.

**Figure 4:**
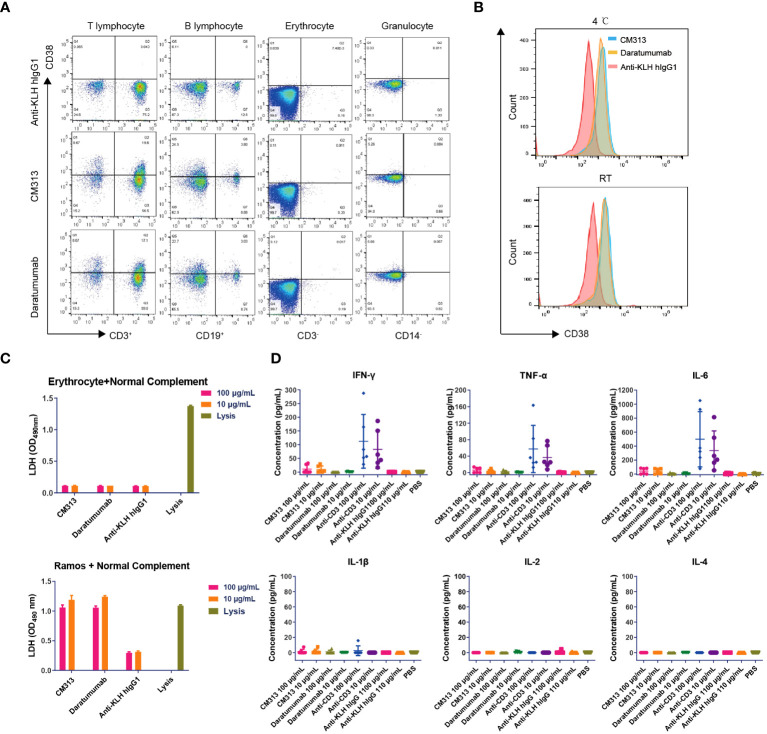
Immunotoxicity of CM313 in human blood. **(A, B)** Binding of CM313 to human blood cells **(A)** and platelets **(B)**. RT, room temperature. **(C)** CDC activity of CM313 on human blood erythrocytes. Ramos cells and cell lysis were used as positive controls. **(D)** Concentrations of IL-4, IL-5, IL-6, IL-1β, TNF-α and IFN-γ after co-incubation of CM313 and healthy human blood. Anti-CD3 was used as positive control and Anti-KLH hIgG1 was used as isotype control. PBS, Phosphate buffered saline.

Previous research has identified CD38 expression in various cell types including erythrocytes, T cells, dendritic cells, and natural killer cells (27, 28), raising questions on whether targeting CD38 induce erythrocyte agglutination and CRS. To address these concerns, we conducted a thorough evaluation of the possible interactions between CM313 and human blood. The human blood cell binding assay revealed that, similar to daratumumab, CM313 exhibited comparable binding to human T cells and B cells, with no apparent binding to human erythrocytes and granulocytes (**Figure 4A**), suggesting that CM313 does not induce human erythrocyte hemolysis and clotting. CM313 also bound weakly to human platelets at both 4°C and room temperature (**Figure 4B**), indicating a low likelihood of CM313 causing platelet activation or clearance. In addition, we investigated whether the binding of CM313 to erythrocytes leads to hemolysis via CDC. It was found that when CM313 was mixed with human erythrocytes in the presence of 5% normal human serum complement, no LDH release from human erythrocytes was observed (**Figure 4C**). Furthermore, we measured serum concentrations of IL-2, IL-4, IL-6, IL-1β, TNF-α and IFN-γ after co-incubation of CM313 with human blood to assess the risk of CRS. Results showed that CM313 did not induce an obvious cytokine release, indicating its low risk for CRS (**Figure 4D**). In summary, no significant toxicities related to CM313 were identified. Moreover, CM313 exhibited minimal reactivity with human blood cells and a low risk of inducing CRS.

## 4 Discussion

We here present the first description of the preclinical characterization of CM313, a novel CD38-targeting mAb. Of note, innovative structure at CDR facilitates CM313 with advanced potentials in affinity superior to another CD38-targeting mAb, daratumumab, without compromising killing activities. CM313 exhibited the capacity to induce CD38^+^ cells eradication through Fc-receptor-dependent mechanisms including ADCC, CDC, ADCP, and apoptosis. The killing effects was comparable with daratumumab but showed more potent CDC activity than isatuximab. CM313 also effectively inhibited the enzymatic activity of CD38. In addition, these *in vitro* findings were substantiated by strong anti-tumor activity observed *in vivo*. Furthermore, the 4-week repeat-dose toxicology study in B-hCD38 mice and cynomolgus monkeys and human blood binding assay collaboratively demonstrated that CM313 was well tolerated with no drug-related clinical signs or off-target risks. Taken together, these findings underscore the rationale for advancing CM313 into clinical development for the treatments of diseases related to CD38-positivte cell depletion.

Daratumumab and isatuximab, both targeting the CD38, are approved therapies for multiple myeloma, yet they exhibit distinct mechanistic profiles (29). Specifically, daratumumab and isatuximab engage different, nonoverlapping epitopes on CD38, with isatuximab targeting an epitope that partially spans the enzyme’s catalytic site, influencing its cyclase and hydrolase activities without obstructing access or altering its structure. In contrast, daratumumab binds to a site remote from the CD38 catalytic region, showing partial inhibition of cyclase activity and an enhancement of hydrolase function (7). Furthermore, daratumumab stands out as a potent inducer of CDC, whereas isatuximab elicits a more modest CDC response. Notably, isatuximab can trigger direct apoptosis in target cells, a process that daratumumab can only initiate through secondary cross-linking (30). In our comparative study of various mechanisms *in vitro*, we conducted a head-to-head comparison between CM313 and daratumumab, revealing that aside from CM313 having a unique CDR sequence and slightly higher affinity, CM313 exhibited comparable tumor cell killing activity, much like daratumumab.

There are also some limitations in this study. In our comparison, the focus was on the differences between CM313 and daratumumab, which may not have fully captured the unique properties of isatuximab, especially its enzymatic inhibition, CDC difference, and direct apoptosis. Isatuximab distinctively inhibits the ectoenzymatic activity of CD38, a feature not observed with daratumumab, which exhibits minimal to no direct inhibition of CD38’s enzymatic function (31). CM313, on the other hand, demonstrates a partial inhibition of this activity, though the specific implications and extent of such inhibition are less comprehensively understood compared to isatuximab. The modulation of CD38 enzymatic activity by isatuximab, and to a lesser degree by daratumumab or CM313, may influence immunomodulatory pathways, notably affecting the activity of natural killer (NK) cells (29). In light of the parallels between CM313 and daratumumab, it has been observed that CM313 exhibits CDC activity akin to that of Daratumumab. The efficacy of daratumumab is known to be modulated by the presence of complement inhibitor proteins, and it is anticipated that CM313’s activity may be comparably affected by these proteins. Isatuximab uniquely triggers direct apoptosis in MM cells, circumventing the need for external cross-linking agents (29), a capability that sets it apart from Daratumumab and CM313. This action unfolds independently of effector cells, such as natural killer cells or macrophages, which are conventionally implicated in mechanisms like ADCC and ADCP. Conversely, CM313 aligns more closely with daratumumab in its action mechanism, both necessitating either cross-linking agents or the involvement of effector cells to incite apoptosis in MM cells. This delineates a critical distinction between CM313 (alongside daratumumab) and isatuximab, with the latter’s capacity for direct apoptosis induction potentially offering therapeutic benefits, especially in contexts where effector cell presence or functionality is diminished (32).

In a comparative analysis of clinical outcomes within an identical patient population, the CANDOR Phase III trial (daratumumab with carfilzomib and dexamethasone, dara+Kd) and the IKEMA Phase III trial (isatuximab with carfilzomib and dexamethasone, isa+Kd) were both directed at patients with relapsed, refractory MM who had undergone 1-3 prior lines of therapy (29). The findings revealed that the regimen dara+Kd, as opposed to Kd monotherapy, achieved an overall response rate (ORR) of 84% compared to 75%, complete response (CR) rates of 29% versus 10%, MRD negativity rates of 18% versus 4%, and a median progression-free survival (mPFS) of 28.6 months against 15.2 months (HR: 0.59; 95% CI: 0.45-0.78, p<0.0001). Similarly, the isa+Kd combination versus Kd alone resulted in ORRs of 87% versus 84%, CR rates of 44% versus 29%, MRD negativity rates of 34% versus 15%, and mPFS of 35.7 months versus 19.2 months (HR: 0.58; 95% CI: 0.42-0.79). These outcomes suggest a comparable clinical efficacy between daratumumab and isatuximab in the combination therapies, with both significantly benefiting patients. This underscores that CD38-targeting antibodies, despite their varied mechanisms of action, can offer distinct clinical advantages to patients.

CM313 exhibits broad but heterogeneous activity against CD38^+^ cells, including MM, Burkitt’s lymphoma, diffuse large B lymphoma, acute B lymphoid leukemia and acute T lymphoid leukemia cell lines. The most potent cell-killing activity observed in MM cell lines and Daudi cells (**Figure 2A**). This variance could potentially be attributed to the heterogeneity of CD38 expression among hematologic malignancies (20). Previous studies have highlighted that CD38 expression in MM is typically high and homogenous, contributing to durable therapeutic responses (7). Conversely, in other hematologic malignancies, such as chronic lymphocytic leukemia, CD38 expression is more variable, with CD38 positivity defined as minimal expression of 20% (33, 34). Thus, this study provides valuable insights into the specific patient populations that may benefit most from CM313 treatment. On the other hand, CD38-targeting mAbs, in combination with IMiD, have demonstrated profound anti-cancer activity with manageable safety profiles in relapsed/refractory and newly diagnosed MM patients (17, 35). In accordance with this, CM313 has shown synergistic killing activity with lenalidomide in the MM.1R tumor (**Figure 3D**). Further mechanistic investigations confirmed that IMiDs cause loss of Ikaros, leading to upregulation of CD38 surface expression on MM cells (36). The mechanistic insights provided rationales for combination therapy involving CM313 and IMiDs. As demonstrated, CM313 exhibited potent anti-tumor activity *in vivo* against MM and lymphoma, both as a standalone alone and rational combination treatments in the current study.

Given broad spectrum of CD38 expression in immune cells, especially the antibody-secreting plasma B cells, CD38 appears to also play essential roles in processes of inflammation during autoimmunity (8). Except for hematological tumors, CD38-targeting therapy, as one of the major discussed immune cell-depleting based strategies, has also been investigated and practiced in clinical trials of a wide range of diseases including SLE (24, 37), ITP (38) and IgA nephropathy (IgAN) (39, 40). Such progressed applications of anti-CD38 mAbs shed new insights to treatment of expanded types of autoimmune indications via clinical use of CM313.

CM313, daratumumab and isatuximab bind to CD38 with high affinity only in human and chimpanzee, but not in cynomolgus monkey, mouse, rat, rabbit or dog. Theoretically, all three of these mAbs lack relevant animal species for both preclinical pharmacokinetic studies and safety evaluations. Nevertheless, in case of preclinical safety evaluation, the repeat toxicity study of CM313 was designed under similar manners to the cynomolgus monkey inspection of isatuximab, with the highest administered dose (206 mg/kg) being twice the amount of isatuximab (30, 41). The results demonstrated the safety of CM313 formulation and no off-target toxicity. Furthermore, we validated its safety in human CD38 transgenic mice in a four-week repeated-dose toxicity study, and the NOAEL was 300 mg/kg. Tissue cross-reactivity results of CM313 were also consistent with those of daratumumab (42).

In conclusion, CM313 represents a novel CD38-targeting mAb exhibiting potent pathogenic CD38^+^ cell-killing activity. The extensive preclinical evaluation results support the advancement of CM313 into clinical trials for CD38^+^ cell depletion and AIDs. Considering especially the trends of current practice of CD38-targeting therapies, CM313 possesses great potential in the treatment of autoimmune diseases where expanding clinical demand remains far unmet. CM313 is currently being evaluated in clinical trials at phase I for the treatment of RRMM (NCT04818372), and phase I/II for SLE (NCT05465707) as well as phase II for ITP (NCT05694767). Meanwhile, the encouraging preliminary data have also been selected to present at the 28th Annual Meeting of the European Association of Hematology (EHA).

## Data availability statement

The original contributions presented in the study are included in the article/supplementary material. Further inquiries can be directed to the corresponding author.

## Ethics statement

The studies involving humans were approved by Hospital Ethics Committee (20212002-C221). The studies were conducted in accordance with the local legislation and institutional requirements. The participants provided their written informed consent to participate in this study. The animal study was approved by Association for Assessment and Accreditation of Laboratory Animal Care International (AAALAC). The study was conducted in accordance with the local legislation and institutional requirements.

## Author contributions

WL: Conceptualization, Formal analysis, Investigation, Methodology, Project administration, Validation, Writing – original draft, Writing – review & editing. JY: Conceptualization, Data curation, Formal analysis, Investigation, Methodology, Project administration, Supervision, Validation, Writing – review & editing. KS: Data curation, Investigation, Validation, Visualization, Writing – review & editing. QS: Investigation, Methodology, Validation, Writing – review & editing. YL: Investigation, Validation, Writing – original draft, Writing – review & editing, Visualization. YH: Investigation, Validation, Writing – review & editing, Writing – original draft, Visualization. YW: Investigation, Methodology, Validation, Writing – review & editing. GX: Conceptualization, Formal analysis, Investigation, Methodology, Resources, Writing – review & editing. CW: Conceptualization, Formal analysis, Investigation, Methodology, Resources, Writing – review & editing. BC: Conceptualization, Funding acquisition, Investigation, Project administration, Resources, Supervision, Writing – review & editing.
